# Treating Adrenal Tumors in 26 Patients with CyberKnife: A Mono-Institutional Experience

**DOI:** 10.1371/journal.pone.0080654

**Published:** 2013-11-20

**Authors:** Jing Li, ZhaoRong Shi, Zhen Wang, Zhibing Liu, Xinhu Wu, Zetian Shen, Bing Li, Yong Song, Xixu Zhu

**Affiliations:** 1 Jingling Hospital, Department of Radiotherapy Center, Nanjing University School of Medicine, Nanjing, China; 2 Jingling Hospital, Department of Radiotherapy Center, Nanjing, China; 3 Jingling Hospital, Nanjing, China; Virginia Commonwealth University School of Medicine, United States of America

## Abstract

**Background:**

CyberKnife (*CK*) is a novel stereotactic radiosurgery system for treating tumors in any part of the body. It is a non-invasive or minimally invasive tumor treatment modality that can deliver high doses of spatially precise radiation and minimize exposure to neighboring healthy tissues or vital organs. The purpose of this study was to investigate the safety and efficacy of *CK* in the treatment of adrenal tumors.

**Methods and Results:**

We performed a retrospective analysis of 26 patients with adrenal tumors who had been treated with *CK* in the radiotherapy center of our hospital between March 2009 and March 2012. Eight patients had primary adrenal tumors and 18 patients had metastatic adrenal tumors. In addition to *CK*, 4 patients received chemotherapy and 2 patients received immunotherapy. The average tumor volume was 72.1 cm^3^ and the prescribed radiation dosage ranged from 30 to 50 Gy and was fractionated 3 to 5 times with a 58% to 80% isodose line. Abdominal *CT* was performed between 1 to 3 months after the *CK* treatment to evaluate the short-term efficacy with follow-up examinations once every 3 months. Three patients had complete remission, 12 patients had partial remission, 5 patients had stable disease, and 6 patients had progressive illness. The effective rate of pain relief was 93.8% and the disease control rate was 77% with a median overall survival of 17 months and a median progression-free survival of 14 months. Treatment Related toxicity was well-tolerated, but preventative measure need to be taken for radiation enteritis.

**Conclusions:**

*CK* is safe and effective for treating adrenal tumors with few adverse reactions. Nonetheless, its long-term effects requires further follow-up.

## Introduction

Adrenal tumors are mostly treated through surgery-based comprehensive therapy. However, the diverse and atypical clinical manifestations often prevent timely diagnosis and treatment. Additionally, patient age, tumor metastasis, contraindications to surgery, and relapses affect treatment efficacy. As a result, radiation therapy plays an important role in the treatment of adrenal tumors. Conventional radiation therapy often targets a large spatial region and requires long durations of radiation exposure. The maximum radiation dosage is limited by the toxicity to surrounding healthy tissue and can often result in disease progression or recurrence, ultimately leading to treatment failure.

Stereotactic radiotherapy allows tumor treatment with high dosage and low fractionation, thereby overcoming the dosage limitations of conventional radiation therapy and improving local control rate [1-5]. CyberKnife (CK) is one type of stereotactic radiosurgery system, employing real-time image guidance and a synchronized respiratory tracking system to deliver high dosages of hypofractionated radiation dynamically to the tumor with spatial precision that can reach the sub-millimeter scale [6]. Due to improved conformation and precision, a lethal dose of radiation can be delivered to the tumor while the surrounding normal tissues or vital organs are radiated with lower dosages and exposure ranges, leading to fewer side effects and improved safety. Our hospital used *CK* therapy for the treatment of adrenal tumors in 26 patients between March 2009 and March 2012. The goal of this study was to assess the safety and efficacy of *CK* therapy in the treatment of adrenal tumors by analyzing the tumor control rate and adverse reactions.

## Materials and Methods

### Patient Information

Retrospective analysis was performed on data of patients with adrenal tumors treated between March 2009 and March 2012. Prior to treatment, all patients underwent pertinent studies (including the head magnetic resonance imaging (MRI), chest and abdominal computed tomography (CT), electrocardiogram (ECG), routine blood tests, blood chemistry panel, and tumor markers). The patients’ conditions were comprehensively assessed by radiologists and urologists, and patients and their families were informed of possible adverse effects during the perioperative and postoperative course of treatment. The study have been approved by the authors' institutional (Jiangsu institute of Medical Ethics). All patients signed an informed consent for *CK* treatment. Medical charts, imaging reports, and films were cross-reviewed by 3 principal investigators.

### Radiotherapy

Pre-treatment preparation: 19 patients underwent *CT*-guided transdermal adrenal puncture and implantation of gold fiducials. An 18GPTC needle was used to puncture the selected points. After *CT* confirmation of placement of the needle within the tumor or nearby regions, the needle core was removed and forceps were used to transfer the gold fiducials from the end of the trocar into the trocar cavity. Subsequently, the needle core was used to move the gold fiducial towards the predetermined location within the tumor tissue at the front end of the trocar. Ideally a minimum of three markers have been implanted to allow for correction of translational and rotational target motions. The minimum distance between each marker should be greater than 2 cm and the angle should be greater than 15 degrees. After markers placement, a planning *CT*-scan was used to confirm the location of the gold fiducials and to rule out complications such as bleeding. In general, gold fiducials migration occurred within 1 week after implantation. Therefore, additional *CT*-scan should be taken at least 7 days after markers implantation to reconfirm positioning [7]. 

Positioning and planning *CT*-scan: patients were positioned and stabilized using vacuum pads. Imaging was performed with Sensation 16 *PET/CT* (Siemens, Germany) with a slice thickness of 1 mm. The upper and lower limits of the imaging spanned 15 cm superior and inferior to the tumor lesions. *CT* images were transmitted by the *DICOM* protocol and then underwent fusion and contouring on a workstation. 

Target outline and treatment plan: The gross tumor volume(GTV) defined as visible tumor. The *PGTV* was formed through extension around the *GTV* by 3 mm in the *x*, *y*, and *z*-axis. *CTV* was defined as GTV + 8 mm and PTV was defined as CTV + 2 mm - 3 mm. Additionally, the organs at risk (OR) and planning organ at risk volume (*PRV*) were determined. Positional imaging and target outline were transmitted to the *CK SGI* workstation using the *DICOM RT* protocol.

Treatment procedures: a synchronized breathing tracking technique was used in 19 patients with gold fiducials implantation. Based on breathing mechanics, the frequency and depth can be measured and recorded. Subsequently, this is used to establish a respiratory motion model and a 4D model of the tumor lesions to track their relative position during breathing, thereby permitting dynamic radiotherapy with a moving target. The X-sight Spine Tracking System was used in 7 patients. The spinal segments closest to the target region were used as a location reference. Patients were oriented in the proper position followed by a spine x-ray, and the corresponding vertebrae were located through automated software, thereby indirectly determining the exact location of the tumor. The software analyzed the ROI bony markers and the 6-D error of patient posture. The manipulator automatically corrected the position and orientation to compensate for the positioning error [6]. Dose fractionation and dosage per session were developed based on the patient’s general condition, performance status, tumor size, and location. Therapy consisted of high-dosage, hypofractionated radiation (6-15 Gy/fraction, split into 3-5 times) delivered once per day (break on weekends), resulting in a total dose of 30Gy-50Gy over 3-5 days (Table 1).

**Table 1:** Treatment characteristics of cyberknife for 26 patients with adrenal tumor.

Treatment characteristics	Range	Mean	Median
Total dose (Gy)	30-50	43	45
Fraction (n)	3-5	-	5
BED* (Gy)	48-113	87	-
Maximum dose (Gy)	42-80	61	-
Isodose line (%)	58-80	-	70
Tumor volume coverage (%)	60-96	-	95

BED*: biological effective dose

Treatment evaluation: guidelines set by the Response Evaluation Criteria in Solid Tumors (RECIST) were used to assess the efficacy. Short-term effects, including changes in lesions and possible radiation injuries, were evaluated using abdominal *CT* imaging that was performed two months after *CK*. Toxicity associated with radiation was assessed in accordance with guidelines set by the National Cancer Institute-common toxicity criteria. Subsequently, *CT* imaging was performed once every three months to evaluate for changes in lesion size. Based on patients’ will and economic factors, tumor markers were not tested. The endpoint of our evaluation was the subsidence of symptoms, tumor local control rate, and radiation injury.

Statistical analysis: OS and PFS were expressed using the Kaplan-Meier survival curves. Comparison of OS was assessed by the log-rank test. Univariate Cox analysis was used to assess the possible predictive factors associated with OS. Due to the small number of subject, a multi-variable analysis was not conducted. All statistical analyses were performed using the SPSS 16.0 software.

## Results

The clinical information of the 26 patients (17 males and 9 females) who underwent *CK* treatment is summarized in Table 2. The median age was 64 years (ranged from 46 to 81 years). We have a complete staging of each patient based on the seventh edition of the Cancer Staging Manual of the American Joint Committee on Cancer. Diagnoses in 22 patients were confirmed histologically or cytologically. Four patients refused biopsy, and confirmation of adrenal masses was performed through *CT* imaging. Using the *ECOG* performance status, the activities of daily living of all patients were evaluated based on a score of 0 to 3. All patients had normal blood tests and liver function tests. Three patients had primary lung cancer with brain and adrenal metastases, and two patients had bone metastases. One patient received three *CK* treatments for metastatic lesions in the adrenal glands, liver, and pancreas after undergoing left renal pelvis cancer surgery. During *CK* treatment, two patients with primary renal cell carcinoma and postoperative metastasis received oral sorafenib as a combined chemotherapy, two patients with primary lung cancer and metastasis received intravenous chemotherapy, and interferon and interleukin immunotherapy was used in one patient with renal cell carcinoma and one with postoperative metastasis.

**Table 2:** Patient demographics and clinical characteristics.

Characteristics		Primary tumor	Metastatic tumor
Total no. patients=26	No. (%)	No. (%)	No. (%)
Sex			
Male	17(65.4)	5(19.2)	12(46.2)
Female	9(34.6)	3(11.5)	6(23.1)
Age(years)			
40-49	4(15.4)	1(3.8)	3(11.5)
50-59	5(19.2)	3(11.5)	2(7.7)
60-69	5(19.2)	2(7.7)	3(11.5)
70-79	8(30.8)	2(7.7)	6(23.1)
80-89	4(15.4)	0	4(15.4)
Performance status			
0	2(7.7)	2(7.7)	0
1	11(42.3)	4(15.4)	7(26.9)
2-3	13(50.0)	2(7.7)	11(42.3)
Histology			
Adenocarcinoma and Squamous cell carcinoma	7(26.9)	0	7(26.9)
Pheochromocytoma	2(7.7)	2(7.7)	0
Neuroendocrine carcinoma	3(11.5)	2(7.7)	1(3.8)
Clear cell carcinoma	4(15.4)	0	4(15.4)
Urothelium carcinoma	6(23.1)	0	6(23.1)
Uncertainty	4(15.4)	4(15.4)	0
Tumor diameter(cm)			
1-5	5(19.2)	2(7.7)	3(11.5)
>5	21(80.8)	6(23.1)	15(57.7)
Tumor location			
Left adrenal gland	15(57.7)	4(15.4)	11(42.3)
Right adrenal gland	11(42.3)	4(15.4)	7(26.9)

The median survival was 17 months (ranged from 6 to 40 months). Five patients with adrenal metastasis had died at the time of data analysis (one patient with primary lung cancer died from respiratory failure while the remaining four patients died from disease progression). These five *CK* patients had pre-treatment *ECOG* scores of 2 to 3, and lesions in two patients were stable after treatment while 3 patients had distant metastases. There were no treatment-related deaths. Upon completion of *CK* treatment, 3 patients received additional stereotactic radiotherapy for distant metastases, 2 patients received systemic intravenous chemotherapy, and 4 patients received oral chemotherapy.

**Figure 1:**
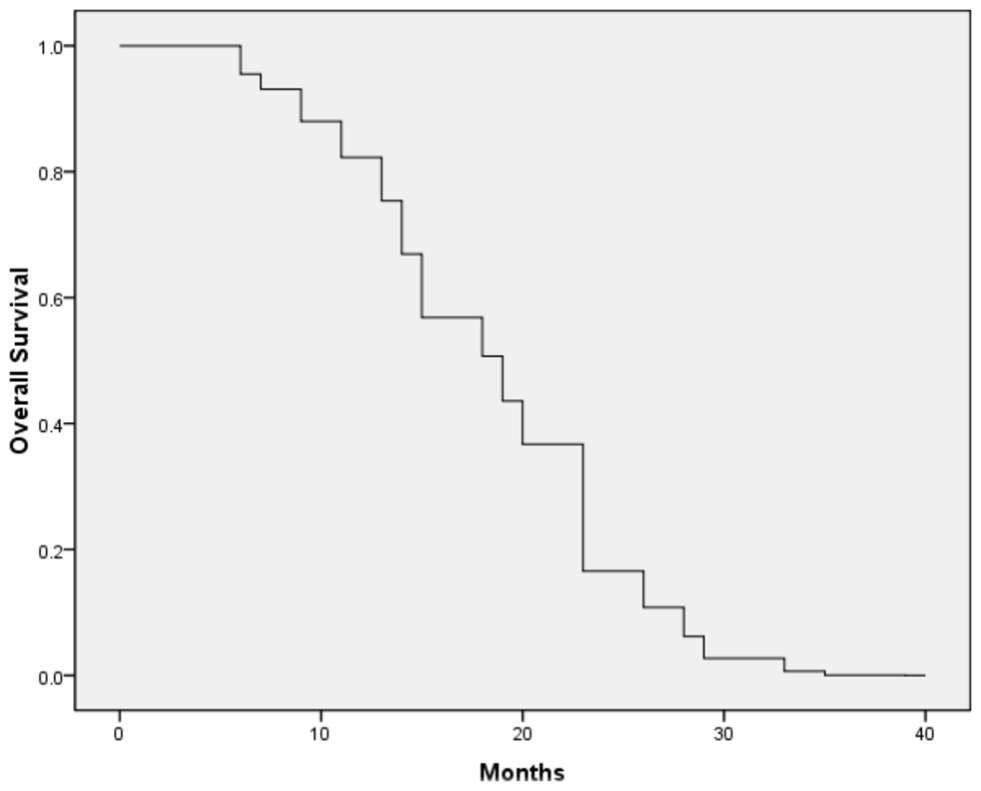
Kaplan-Meier plots. Overall survival for all patients (n=26).

**Figure 2:**
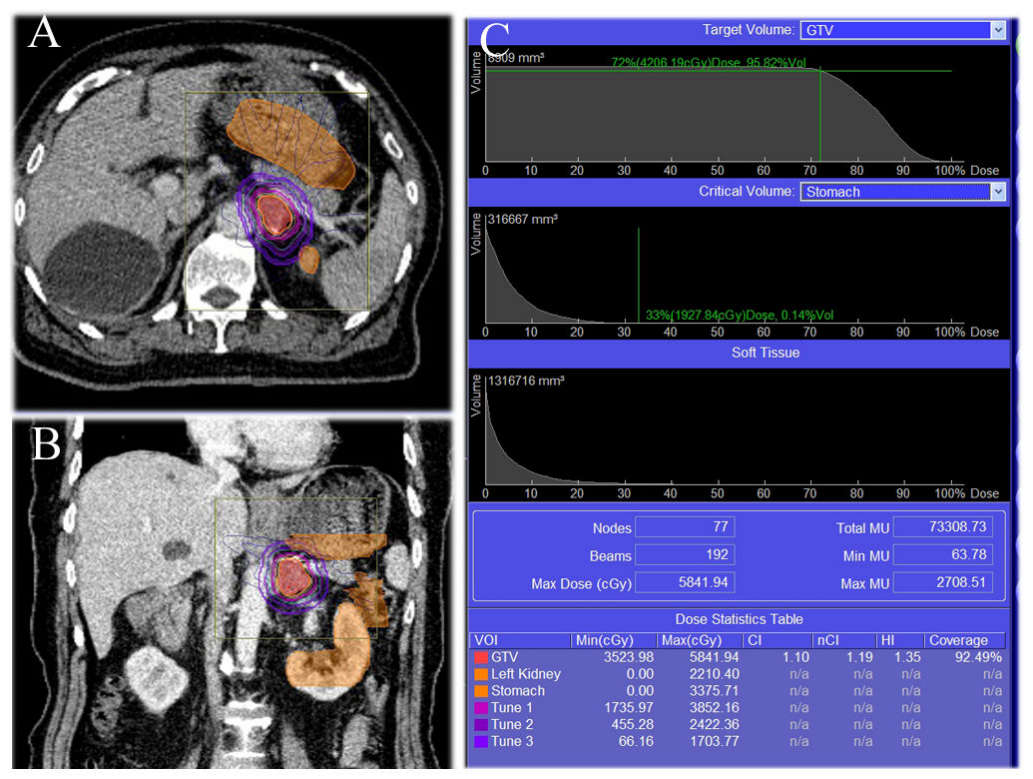
Treatment results and dosimetry plots of one case. A 80 years old male patient with squamous cell carcinoma involving the right lung with isolation metastasis in the left adrenal, with tumor size of 3.6×1.7 cm. (A) The DVH of CK treatment shows tumor in the high-dose region, meanwhile surrounding normal tissues in the low-dose region. (B) The OAR including the left kidney, stomach and very small part of the intestinal tract. (C) Therapy consisted of high-dosage, hypofractionated radiation (15 Gy/fraction, split into 3 times) delivered once per day, resulting in a total dose of 450Gy. The Max and Min dosage(Gy) of GTV was 58.4 and 35.2 respectively. Tumor volume coverage was 92.5%.The Max dosage(Gy) of left kidney and stomach was 22.1 and 33.7 respectively.

**Figure 3:**
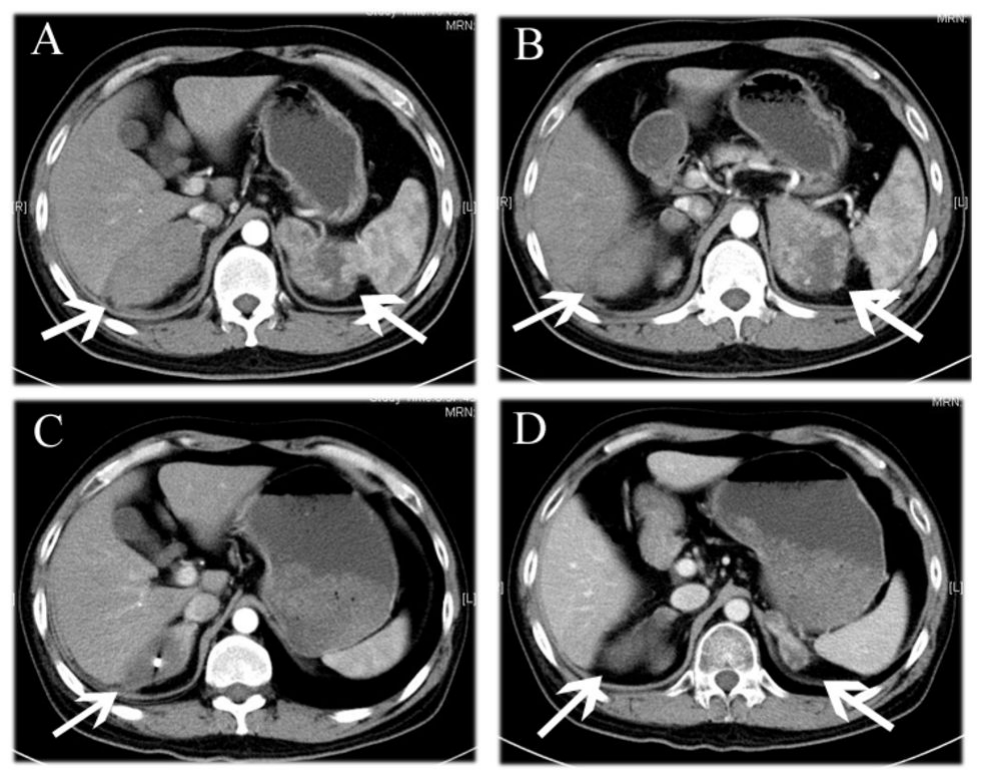
Comparison before CyberKnife treatment with that at 2 months after treatment. A 40 years old male patient with adenocarcinoma involving the right lung with metastases in the left and right adrenal after 4 cycles of chemotherapy. (A, B) Abdominal transverse enhanced CT scanning shows bilateral adrenal metastasis. (C, D) Abdominal transverse enhanced CT at 2 months after CK treatment shows bilateral metastases were significantly reduces with the previous. The patient had varying degrees of lower back and abdomen swelling pain before treatment, the symptom were relieved at the time when the patient was followed up.

Of all the cases, three patients with small lesions achieved complete remission (CR), 12 had partial remission (PR), 5 had stable disease (SD), and 6 had progressive disease (PD)(Table 3). The overall effective rate was 58% and the disease control rate was 77%. Out of 6 patients with progressive disease (PD), 4 had lesions reduced to PR but with distant metastases while 2 patients had local tumor relapses. Based on the subgroup analysis, the effective rates and local control rates in patients receiving a biological effective dose (BED) ≥100 Gy was higher than those receiving <100 Gy (effective rates: 67% vs. 59%; local control rates: 100% vs. 82%). Effective rate in patients with total dosages ≥45Gy was higher than in patients receiving <45Gy (60% vs. 45%) while the local control rate was not significantly different (73% vs. 73%; Table 3). The overall median survival was 1 year in 62% of patients (95% CI, 90.1-100%) and 2 years in 30% of patients (70-98.3%) (Figure 1). Survival-associated factors were assessed by univariate analysis, suggested that metastatic tumors significantly affected the patients’ *OS* (*P* = 0.018) (Table 4; see Figure 2 and 3 for planning and results of the CK treatment). 

**Table 3:** Outcome and death of cyberkinfe for 26 patients with adrenal tumor.

Subgroup		RR*	DCR*	Death
	n	No. (%)	No. (%)	No. (%)
Total	26	58(15/26)	77(20/26)	19(5/26)
Primary tumor	8	88(7/8)	88(7/8)	0
Metastatic tumor	18	44(8/18)	72(13/18)	28(5/18)
Sex				
Male	17	53(9/17)	71(12/17)	18(3/17)
Female	9	67(6/9)	89(8/9)	22(2/9)
BED (Gy)				
<100	17	59(10/17)	82(14/17)	18(3/17)
≥100	9	67(6/9)	100(7/7)	22(2/9)
Total dose(Gy)				
≥45	15	60(9/15)	73(11/15)	20(3/15)
<45	11	45(5/11)	73(8/11)	18(2/11)

RR: response rateDCR: disease control rate

**Table 4:** Univariate analysis for survival.

	RR	95%CI	P
Age (≥64years vs.<64years)	0.788	0.301-2.066	0.628
Tumor diameter(≥5cmvs.<5cm )	2.067	0.604-7.072	0.247
Tumor location( left vs. right )	1.431	0.469-4.365	0.528
Performance status( ≥2 vs. <2 )	0.850	0.279-2.595	0.776
Tumor nature( primary vs. metastatic )	4.079	1.275-13.050	0.018*
Sex( female vs. male)	0.125	0.037-0.422	0.001**

* The metastatic tumors play a great role in the death rate

** In this study, most of the patients were male(most of them have metastatic adrenal tumors). So the sex was found not to be significantly associated with better survival

Sixteen patients had varying degrees of lower back swelling pain prior to CK treatment. Based on the Budzynsk pain scale, symptoms of pain in 12 patients (75%) disappeared by the end of treatment, 3 patients (18.85%) reported pain relief, and 1 patient reported ineffective pain management. The effective rate of pain relief was 93.8% .

Adverse events associated with treatment are shown in Table 5. The most common side effects were grade 1/2 fatigue and gastrointestinal discomfort. No serious bone marrow toxic reactions were detected while grade 1/2 bone marrow suppression was common. Treatment in one patient was interrupted due to old age, large lesion size, long duration of single treatment session, and treatment was resumed the following day. According to the RTOG grading criteria, one patient had grade 2 radiation enteritis two weeks after treatment and was discharged after symptomatic treatment. Grade 3 or 4 radiation injuries did not occur in any of the patients.

**Table 5:** Summary of adverse events from cyberknife-treatment.

	No. of patients (n=26)			
Toxicity	Grade 1-2	Grade 3	Grade 4	Grade 5
Dermatology(rash, pruritus, aridity)	6	0	0	0
GI tract				
Anorexia	18	0	0	0
Nausea/vomiting	10	1	0	0
Diarrhea	4	0	0	0
Chordapsus	1	0	0	0
Hematology				
Anemia	8	1	0	0
Neutropenia	3	2	0	0
Thrombocytopenia	12	2	0	0
Constitutional symptoms				
Myalgia	3	0	0	0
Fatigue	23	0	0	0

## Discussion

The organ predilection of malignant tumor metastasis includes the adrenal glands and ranks fourth worldwide after lung, liver, and bone metastases [8]. In our study, metastatic tumors were present in 18 out of 26 patients (69%). The vast majority of primary adrenal tumors had atypical clinical manifestations. Few patients have early symptoms and screening was not routinely performed. The metastatic tumor may have also metastasized to other organs, minimizing the effect of surgical treatment. Because of their unique anatomical location and the dosage limitation established by neighboring healthy tissue and vital organs, therapeutic effects are often unsatisfactory. Therefore, treatment with *CK* may provide a more effective therapy.

*CK* treatment is strongly beneficial in patients with adrenal tumors who cannot undergo surgery or decline surgery or patients who have failed postoperative adjuvant chemotherapy and conventional radiotherapy. *CK* combines the modalities of both radiosurgery and radiotherapy. The linear accelerator of the manipulator can radiate in any direction at a given angle around the patient, providing maximum control over the shape of the isodose line and optimally fit the 3-D conformation of the tumor while avoiding vital organs. When a synchronized breathing tracking system is used, a spatial model of the tumor can be made with respect to respiratory movement. Through instantaneous comparisons and adjustments during treatment, the irradiating direction can be repositioned and corrected in real-time, resulting in spatially precise targeting. As a result, the surrounding healthy tissues and vital organs are exposed to a smaller range and lower dosages of radiation, thereby lowering toxicity, improving safety, and significantly increasing disease control rate and radiobiological effects. Additionally, *CK* is non-invasive and delivers larger single-fraction dosages with fewer fractions, thereby overcoming the limitations of conventional radiotherapy. Compared with conventional 3-D conformal radiotherapy, the average radiation dose is increased by 75% and the average minimum radiation dose is increased by 51%. The mean spatial error is only 0.7 ± 0.3 mm and the accuracy can reach 0.3 ± 0.1 mm [9-11].

Previous studies [12-14] have shown that *CK* treatment is effective in lung and brain tumors, even achieving the same therapeutic effects as surgery. However, little is known about the effectiveness of *CK* in treating adrenal tumors, and this study is exploratory. Of the 26 patients in our study, sixteen patients had varying degrees of lower back swelling pain. Pain symptoms were significantly alleviated after to CK treatment, with an overall effective rate of 93.8%. Pain relief mechanism may include the following aspects: (1) tumor volume was reduced by high doses of spatially precise radiation, relieving the simulation and oppression repressed on the peripheral nerves; (2) reducing or terminating the release of algogenic substance, such as 5-serotonin, bradykinin, prostaglandins etc; (3) micro-thrombosis or fibrosis of cancer blood vessels or peritumoral vascular, preventing algogenic substance through; (4) functional electrophysiological conduction block or degeneration of nerve sheath breaking the pain pathway. Based on the imaging reports, of the 26 patients in our study, 3 achieved *CR*, 12 achieved *PR*, 5 had *SD*, and 6 had *PD*. Of the patients with *PD*, 4 had shrunken lesions and eventually achieved *PR* albeit with distant metastases and localized relapses in 2 patients. The overall effective rate was 58%, and disease control rate was 77%. In our study, metastatic tumors were found in 18 patients. As a result, metastases to other locations or increased metastatic foci were detected in many patients during follow-up. Additionally, 62% of patients were over the age of 60 years. Due to their poor physical health and reluctance to undergo surgery, localized lesions in these patients were not well controlled after several rounds of chemotherapy, and they were referred for *CK* treatment as a result. Due to age, physical status, tumor size and location and other factors, only palliative doses were administered in some patients. Subgroup analyses showed superior local control rate and effective rate in patients with BED ≥100 Gy, suggesting that BED may be important in influencing the therapeutic effect of *CK*. This had been confirmed in previously reported studies [15-17]. The inferior local control rate in patients receiving a total dose of ≥45 Gy is mainly due to tumor histology, size, and location. Lastly, *CK* treatment of adrenal tumors has no established guidelines for total dosage, number of fractions, single-dose amount, and interval. Development of these standards is currently under investigation.

Radiation injury is the main factor limiting dosage and is associated with the range and dosage of radiation [18]. *CK* is spatially precise and can reduce radiation exposure range and dosage of the neighboring healthy tissues and vital organs, leading to a lower incidence of radiation injury. Anatomically, the adrenal gland is adjacent to the kidney, stomach, pancreas, and intestines. Therefore, the delivery of large radiation doses with high spatial precision is particularly important. During the formulation of treatment plans for our patients, strict dose restrictions were made to accommodate the vital organs (i.e. the ipsilateral kidney, spinal cord, and gastrointestinal tract). As a result, only one patient developed acute radiation injury after treatment. Most common adverse reactions include fatigue and gastrointestinal discomfort.

Our preliminary results indicate that *CK* is safe for treating adrenal tumors and has superior short-term efficacy with lower toxicity. Our study can be used to justify further application of *CK* in the treatment of adrenal tumors. Nonetheless, this study is limited by the small patient population in a single center and the short follow-up time. Therefore, long-term efficacy and delayed radiation injuries require further follow-up and analysis.
