# Correction: Treating Adrenal Tumors in 26 Patients with CyberKnife: A Mono-Institutional Experience

**DOI:** 10.1371/journal.pone.0110147

**Published:** 2014-10-02

**Authors:** The PLOS ONE Staff

There is an error in the Affiliations. Affiliations 1, 2, and 3 should list “Jinling Hospital” rather than “Jingling Hospital.” The affiliations should appear as shown here:

Jing Li^2^, ZhaoRong Shi^3^, Zhen Wang^2^, Zhibing Liu^1^, Xinhu Wu^2^, Zetian Shen^2^, Bing Li^2^, Yong Song^3^, Xixu Zhu^2*^

1 Jinling Hospital, Department of Radiotherapy Center, Nanjing University School of Medicine, Nanjing, China

2 Jinling Hospital, Department of Radiotherapy Center, Nanjing, China

3 Jinling Hospital, Nanjing, China
